# Metabolome profile variations in common bean (*Phaseolus vulgaris L.*) resistant and susceptible genotypes incited by rust (*Uromyces appendiculatus*)

**DOI:** 10.3389/fgene.2023.1141201

**Published:** 2023-03-16

**Authors:** Penny Makhumbila, Molemi E. Rauwane, Hangwani H. Muedi, Ntakadzeni E. Madala, Sandiswa Figlan

**Affiliations:** ^1^ Department of Agriculture and Animal Health, School of Agriculture and Life Sciences, College of Agriculture and Environmental Sciences, University of South Africa, Roodeport, South Africa; ^2^ Department of Botany, Nelson Mandela University, Port Elizabeth, South Africa; ^3^ Research Support Services, North-West Provincial Department of Agriculture and Rural Development, Potchefstroom, South Africa; ^4^ Department of Biochemistry, School of Mathematical and Natural Sciences, University of Venda, Thohoyandou, South Africa

**Keywords:** *Uromyces appendiculatus*, rust, *Phaseolus vulgaris*, common bean, metabolomics, LC-MS

## Abstract

The causal agent of rust, *Uromyces appendiculatus* is a major constraint for common bean (*Phaseolus vulgaris*) production. This pathogen causes substantial yield losses in many common bean production areas worldwide. *U. appendiculatus* is widely distributed and although there have been numerous breakthroughs in breeding for resistance, its ability to mutate and evolve still poses a major threat to common bean production. An understanding of plant phytochemical properties can aid in accelerating breeding for rust resistance. In this study, metabolome profiles of two common bean genotypes Teebus-RR-1 (resistant) and Golden Gate Wax (susceptible) were investigated for their response to *U. appendiculatus* races (1 and 3) at 14- and 21-days post-infection (dpi) using liquid chromatography-quadrupole time-of-flight tandem mass spectrometry (LC-qTOF-MS). Non-targeted data analysis revealed 71 known metabolites that were putatively annotated, and a total of 33 were statistically significant. Key metabolites including flavonoids, terpenoids, alkaloids and lipids were found to be incited by rust infections in both genotypes. Resistant genotype as compared to the susceptible genotype differentially enriched metabolites including aconifine, D-sucrose, galangin, rutarin and others as a defence mechanism against the rust pathogen. The results suggest that timely response to pathogen attack by signalling the production of specific metabolites can be used as a strategy to understand plant defence. This is the first study to illustrate the utilization of metabolomics to understand the interaction of common bean with rust.

## 1 Introduction

Common bean (*Phaseolus vulgaris*) is one of the most important human and animal consumable legume worldwide (Vega et al., 2017; Sadohara, 2020). Globally, common bean is cultivated on about 30 million hectares (Mha), while in Africa over 7.5 Mha are cultivated (Mukankusi et al., 2019). Common bean rust, a disease originating from the fungal pathogen *Uromyces appendiculatus* species affects the production of common bean in many production areas by causing between mild and severe damage to plants upon infection (Delgado et al., 2013). The urediniospores of *U. appendiculatus* can survive in winter and can be a source of inoculum in summer when the humidity and temperature are favourable (Gross and Venette, 2001; Mukankusi et al., 2019). The severity of the pathogen may occur in cooler environments with temperatures of about 17°C–20°C and high humidity (Liebenberg et al., 2005). It is difficult to predict yield losses from rust infestation, the loss of yields of up to 100% may occur under severe disease pressure (Lindgren et al., 1995; Singh and Schwartz, 2010). Gross and Venette (2001) also reported that even yield losses of about 6% can have reduced financial gain to farmers growing the crop on a larger scale. Currently, climate changes favour rust spreads in common bean producing areas as spores favourably flow in the air from one area to the other (Alleyne et al., 2008; Singh and Schwartz, 2010). Singh and Schwartz (2010) also added that plant debris left over from the previous season can also be a source of the disease. Pathogen-infected plants exhibit signs of whitish raised spots on the underside of the leaf that enlarge overtime (6 days) and form brownish uredenia (Souza et al., 2008). Uredenia of the pathogen spreads rapidly from 10–22 days post-infection (vegetative—flowering stages), depending on the conditions of the environment (Devi et al., 2020).

Rust races 1, 3, 5 and others were first characterised in other parts of the world (Brazil and United States) and were later observed in Southern Africa, indicating prevalence of numerous races of *U. appendiculatus* (Liebenberg et al., 2005; Aruga et al., 2012). South Africa’s major production areas including Mpumalanga, Free State, North West, Gauteng and KwaZulu-Natal are greatly affected by rust races 1, 3 and 11. Although rust race 11 is not highly prevalent, it may cause greater yield losses when compared to other races (Liebenberg and Pretorius, 2004). The overall yield of common bean genotypes can be determined by the development of physiological characteristics (Rosales et al., 2012). Any interference by stressors such as diseases at critical phenological stages such as the vegetative development stages of the plant (V1–V4), pre-flowering (V5) and flowering (V6) among others can result in reduced yield (Singh and Schwartz, 2010; Odogwu et al., 2017). Therefore, it is important to utilise an array of methods to manage the *U. appendiculatus* pathogen in common bean during these critical stages production areas (Souza et al., 2013).

The management of the pathogen relies primarily on the following strategies: i) application of cultural practices, ii) fungicide or chemical application, iii) biological control and iv) host plant resistance (Obongoya et al., 2010). However, due to the aggressiveness of the pathogen and its constant evolution, controlling the pathogen has been problematic (Breiing et al., 2021). Understanding the molecular and biochemical mechanisms involved in plant-host interaction can aid in the development of efficient pathogen management strategies that can improve productivity (Kalavacharia et al., 2000). Progress in legume improvement strategies has been witnessed through the advancement of next-generation sequencing techniques (NGS) and other high throughput genotyping technologies that have been applied to interrogate plant-host interactions (Rubiales et al., 2011; Tayeh et al., 2015; Janila et al., 2016, 2016). This development has led to more omics studies of common bean in response to *U. appendiculatus* attack (Cooper et al., 2007; Puthoff et al., 2008). For example, methylated and acetylated histones linked to resistance to *U. appendiculatus* were reported in a genome-wide profiling of histone modifications and gene expression in common bean (Ayyappan et al., 2015). In a similar study, *U. appendiculatus* resistant genotypes were reported to mediate antioxidant enzymes, phenolic compounds, and other defence genes in response to rust infection (Omara et al., 2022). However, metabolomic changes in common bean after infection with *U. appendiculatus* have not been addressed yet.

Metabolomic techniques have played a vital role in aiding researchers to identify significant metabolites that contribute to legume improvement (Ramalingam et al., 2015). Profiling of metabolites using LC-MS techniques has been widely utilised to evaluate legume performance under disease pressure (Makhumbila et al., 2022). In a recent study, common bean infected with *Fusarium solani* significantly enriched biosynthesis of amino acids, flavonoid biosynthesis, purine metabolism and other pathways as a pathogen adaptation strategy (Chen et al., 2020). Amino acids and sugars are among metabolite classes that have been found to be up/down regulated in pea infected with *Rhizoctonia solani* (Turetschek et al., 2017). Similar results have been observed in common bean infected with *Fusarium oxysporum* where numerous metabolites were highly enriched after pathogen attack (Chen et al., 2019). Genes of *U. appendiculatus* have been profiled to efficiently understand the genomic characteristics of the pathogen and it was found that the pathogen alters its genes at different growth stages of the plant (Link, 2020). Although the mode of pathogen action has been evaluated, there is a vast knowledge gap on how common bean genotypes respond to *U. appendiculatus* infection at different growth stages. In order to understand the dynamics of breeding for rust resistance in common bean, metabolomics can play a vital role in selecting parental genotypes for breeding programmes by providing a basis for resistance biomarkers. In this study, the aim was to evaluate metabolomic changes that occur in resistant (Teebus-RR-1) and susceptible (Golden Gate Wax) common bean genotypes when infected with pathogenic *U. appendiculatus* fungal races (race 1 and 3) at two-time points (14- and 21- dpi). The functions of the identified metabolites expressed were studied, including their significance to *U. appendiculatus* tolerance at different growth stages.

## 2 Materials and methods

### 2.1 Plant material and treatments

**FIGURE 1:**
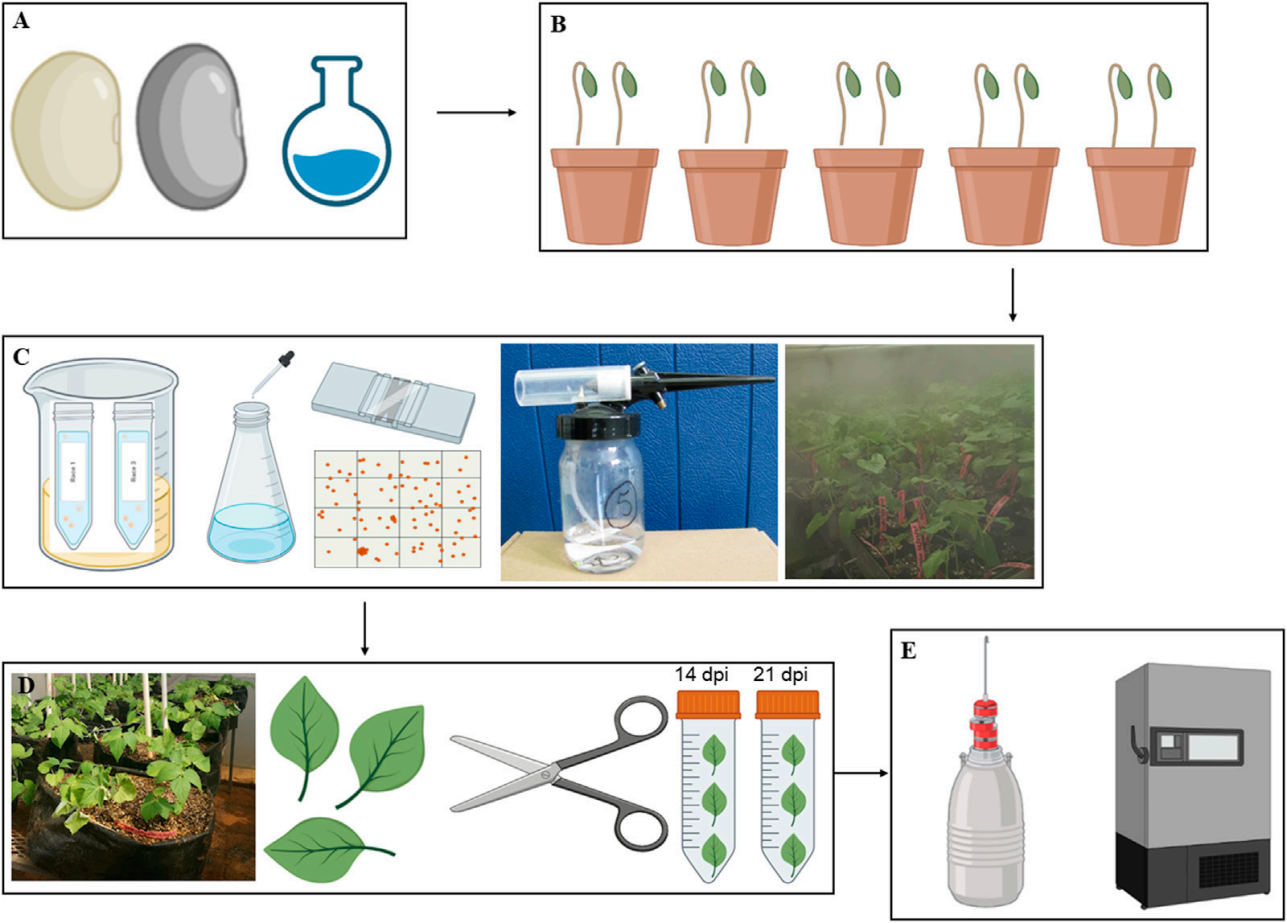
A graphical representation of the methodology workflow from seed treatment and planting **(A and B)**, pathogen preparation and inoculation **(C)**, leaf sample harvesting **(D)** and the storage of harvested material **(E)**.

Seeds of common bean genotypes Teebus-RR-1 (resistant/tolerant) and Golden Gate Wax (susceptible) mapped for rust resistant genes (*Ur-3* and *Ur-6*) were obtained from Agricultural Research Council–Grain Crops Institute (ARC-GCI), Potchefstroom, South Africa. Seeds were surface sterilised using 50% bleach solution (Lindsey et al., 2017), rinsed with sterile water, and grown in 9 cm pots with sterile 30 dm^3^ seedling mix having 50% topsoil and compost, and covered with a vermiculite layer (Figures 1A, B). Inoculation of the respective genotypes was conducted when the leaves were ± $\frac{1}{2}$ − $\frac{3}{4}$ expanded, with five replicates per genotype (Figure 1B).

### 2.2 Fungal material and inoculation

Spores of *U. appendiculatus* races 1 and 3 were provided by ARC-GCI (South Africa) for inoculation purposes. The rust races were previously characterised and collected from isolated common bean infected plants, purified, and were stored in a −80°C ultra-freezer. Purified isolates were then re-hydrated by incubating open cryotubes with the pathogen in a glass beaker with warm water and vermiculite (Figure 1B). The beaker containing the cryotubes with the rust pathogens was sealed with sterile cling wrap plastic and left for 12 h at ±18°C. An aqueous soap suspension of Tween 20 (pure liquid soap) with 5 drops per litre of tap water (rust requires rust Ca^2+^ and Mg^2+^ ions) was prepared and the rust in cryotubes mixed thoroughly with the suspension. The concentration of rust spores was adjusted to 2.5 × 10^4^ spores per ml using a hemacytometer (Figure 1C) with spore counts repeated four times to obtain 100% germination of spores (Montejo Dominguez et al., 2022).

Plants were spray inoculated on the leaves (the underside leaf targeted) at a low pressure using a compressor attached to a bottle with a spray gun in a disinfected confined booth. The inoculated plants were then left to dry for about 20–30 min and later placed in a dew chamber (Figure 1C) with 95%–100% relative humidity and temperatures of ±18–± 20°C for 48 h. The control experimental plants were mock inoculated with distilled water and subjected to the same treatment as the rust inoculated experimental plants. The inoculated plants were kept in different greenhouse compartments with 28/14°C day/night temperatures with relative humidity of 75% (Singh and Gupta, 2019). The plants were transferred from the 9 cm pots to 50 L black planting bags (Figure 1D) with sterile oxidic soil. The scoring of rust infection severity based on pustule size (Table 1) was conducted at 14- and 21- dpi as described by Hurtado-Gonzales et al. (2017).

**TABLE 1:** Uredenia leaf score criteria used for scoring *U. appendiculatus* severity in common bean plants after inoculation.

Score	Description
1	No symptoms
2	Necrotic fleeks or spots without uredenia
3	Uredenia less than 300 µm in diameter
4	Uredenia 300–499 µm in diameter
5	Uredenia 500–799 µm in diameter
6	Uredenia more than 800 µm in diameter

### 2.3 Harvesting and metabolite extraction

Infected (race 1 and 3) and non-infected (controls) leaf material was harvested from the two genotypes at 14- and 21-dpi, representing flowering and pre-flowering stages, respectively (Dann and Deverall, 1996; Devi et al., 2020). The harvested samples were snap frozen with liquid nitrogen and stored in a −80°C ultra-freezer prior to further analysis (Figure 1E). Leaf samples were then weighed (20 mg) and ground into powder in liquid nitrogen using mortar and pestle and extracted using the methanol extraction method consisting of 1.5 mL (1:75 m/v) of 70% LC/MS grade methanol (Merck, Darmstadt, Germany). The extracted samples were vortexed for 30 s, sonicated for 10 min and centrifuged for 5 min at 5,100 rpm (Thermo Fisher, Johannesburg, South Africa). The supernatant was collected and filtered using nylon filters (0.22 µm) into glass vials containing 500 µL inserts (Agela Technologies, Tianjin, China). Three replicates per sample group were prepared for analysis and extracts were stored at 4°C prior to metabolite profiling.

### 2.4 LC-MS metabolite analysis

Infected (race 1 and 3) and non-infected common bean leaf extracts were subjected to analysis on a liquid chromatography-quadrupole time-of flights tandem mass spectrometry instrument (LCMS-9030 qTOF, Shimadzu Corporation, Kyoto, Japan) for quantification of metabolites at different time intervals. A Shim-pack Velox C18 column (100 mm × 2.1 mm with a 2.7 µm particle size) was used for chromatographic separation at 55°C (Shimadzu Corporation, Kyoto, Japan). An injection volume of 3 µL was used for all samples and were run on a binary mobile phase including solvent A: 0.1% formic acid in Milli-Q HPLC grade water (Merck, Darmstadt, Germany) and solvent B: UHPLC grade methanol with 0.1% formic acid (Romil Ltd., Cambridge, United Kingdom). Chromatographic analysis was done using qTOF high-definition mass spectrometer that was set to negative electrospray ionisation for data acquisition. Parameters set included nebulization, interface voltage (4.0 kV), interface temperature (300°C), dry gas flow (3 L/min), detector voltage (1.8 kV), heat block (400°C), DL (280°C) and flight tube (42°C) temperatures. Ion fragmentation was achieved using argon gas for collision with an energy of 30 eV and 5 eV spread (Ramabulana et al., 2021).

### 2.5 Multivariate data analysis

Data pre-processing was done using XCMS, with HPLC/UHD-qTOF parameters using the centWave feature detection method, maximum threshold of 15 ppm, a signal to noise ratio of 6, prefilters for intensity and noise at 100 and 3. The retention time correction method was obiwarp with profStep, while the alignment minimum fraction of samples was 0.5 and a 0.015 m/z width. Kruskal–Wallis statistical test was applied to the data that resulted in a feature table with 11,315 characteristics. The feature table was exported to SIMCA version 17.0 software, normalised and pareto scaled prior to model application. Principal Component Analysis (PCA) and Orthogonal Projection to Latent Structures—Discriminant Analysis (OPLS-DA) models were applied to the data.

### 2.6 Metabolite annotation, relative quantification and pathway analysis

MzMine v2.3 was used for data visualisation, chromatogram deconvolution, MS^1^/MS^2^ building, isotope grouping, alignment, filtering and gap filling (Pluskal et al., 2010). The resulting mascot generic format (mfg.) file and metadata for the respective treatments were processed on GNPS online. Libraries used for spectral search included GNPS, ChEBI, HMBD, DRUGNANK, FooDB, and SUPNAT (Wang, 2016). Metabolites were matched to GNPS linked databases and were also putatively annotated or verified through searches in compound databases using their peak mass and isomeric SMILES. Databases including KEGG compound, KNApSAcK, Chemspider, ChEBI, PubChem, and Dictionary of Natural Products. Annotations were further confirmed through literature search of related studies. Metabolite concentrations were used for an overview of metabolomic pathways that were enriched. Overrepresentation with a hypergeometric test and KEGG metabolite pathway for *Arabidopsis thaliana* (thale cress) was used for pathway analysis in MetaboAnalyst v5.0.

## 3 Results

### 3.1 Phenotypic evaluation of common bean post inoculation with *U. appendiculatus*

**FIGURE 2:**
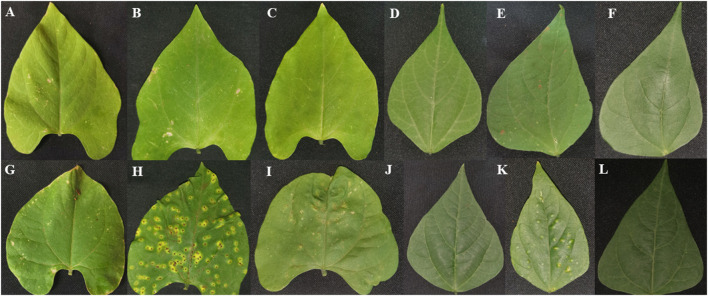
Phenotypic leaf evaluation of Teebus-RR-1 and Golden Gate Wax genotypes in response to *U. appendiculatus* infection. **(A)** Teebus-RR-1 control mock inoculated at 14 dpi, **(B)** Teebus-RR-1 race 1 inoculated leaf at 14 dpi, **(C)** Teebus-RR-1 race 3 inoculated leaf at 14 dpi, **(D)** Teebus-RR-1 control mock inoculated at 21 dpi, **(E)** Teebus-RR-1race 1 inoculated at 21 dpi and **(F)** Teebus-RR-1 race 3 inoculated at 21 dpi. **(G)** Golden Gate Wax control mock inoculated at 14 dpi, **(H)** Golden Gate Wax race 1 inoculated at 14 dpi, **(I)** Golden Gate Wax race 3 inoculated leaf at 14 dpi, **(J)** Golden Gate Wax control mock inoculated at 21 dpi, **(K)** Golden Gate Wax race 1 inoculated at 21 dpi and **(L)** Golden Gate Wax race 3 inoculated at 21 dpi.

Plant leaves of Teebus-RR-1 and Golden Gate Wax common bean genotypes were evaluated phenotypically for their response to *U. appendiculatus* race 1 and 3 throughout the experiment (Figure 2; Table 1). The resistant genotype Teebus-RR-1 exhibited no symptoms of infection by race 1 and 3 at 14 dpi (Figures 2B, C). Similar phenotypic observations were observed at 21 dpi on secondary leaves, after the primary leaves had matured and fallen off (Figures 2E, F). Golden Gate Wax had the highest number of pustules when infected with rust races 1 and 3 at the two time points post infection (Figures 2H, I). Secondary leaves of the susceptible genotypes exhibited a continuous spread of infection with rust race 1 at 21 dpi (Figure 2K) while race 3 had no visible pustules (Figure 2L). Leaf lesions, necrosis and wilting were among symptoms that were prevalent on susceptible genotype Golden Gate Wax at 14 and 21 dpi compared to the control mock inoculated leaves (Figures 2G, J).

### 3.2 Comprehensive analysis of common bean metabolites

Untargeted metabolite profiles of genotypes Teebus-RR-1 and Golden Gate Wax were analysed using LC-MS at 14- and 21- dpi in response to *U. appendiculatus*. The Principal Component Analysis (PCA) model provided the virtual analysis of the effects of *U. appendiculatus* treatments on common bean, revealing clustering on genotypes, races and time intervals post infection. The PCA results showed clustering between the two genotypes (Supplementary Figure S1). Although the PCA score plot showed differential sample clustering with a separation of genotypes (indicating differential metabolite profiles), Othorgonal (OPLS-DA) was computed to allow prediction of variations, consequently allowing identification of potential biomarkers (Tugizimana et al., 2013). The OPLS-DA results revealed similar clustering patterns between the two genotypes (Supplementary Figure S2), reflecting the differences in metabolite profiles between the genotypes.

**FIGURE 3:**
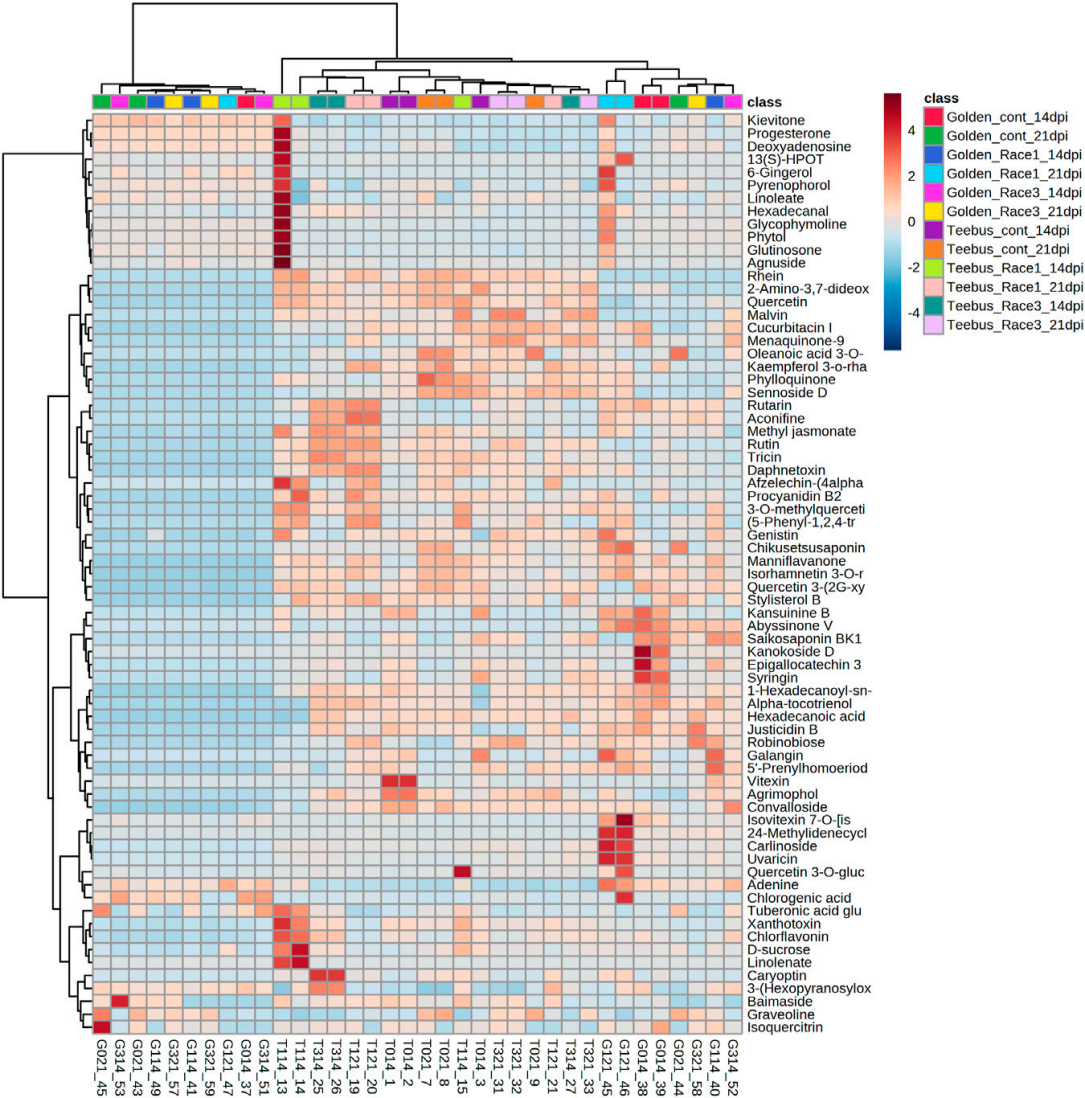
Heatmap of metabolite concentrations of common bean leaves of resistant genotype Teebus_RR_1 and susceptible genotype Golden Gate Wax infected with *U. appendiculatus* races 1 and 3 at two time points (14- and 21- dpi). Class colours represent the experimental treatments corresponding to samples, while numbers show the up/down regulation metabolites identified.

A total of 71 known metabolites were identified to be present in both genotypes at varying concentrations for the different treatments and a heatmap with the 33 metabolites were found to be significant (Table 2). Interestingly, excessive metabolite changes were observed between treatments of the susceptible genotype Golden Gate Wax compared to the resistant genotype Teebus-RR-1 that had slight or limited metabolite changes when subjected to *U. appendiculatus* infection at the different time points. An example, afzelechin-(4alpha->8)-afzelechin, (5-Phenyl-1,2,4-triazol-3-yl) urea, tuberonic acid glucoside, xanthotoxin, chlorflavonin, D-sucrose and linoleate were highly concentrated in Teebus-RR-1 samples infected with rust race 1 at 14 dpi (T114) while there were low concentrations of these metabolites in Golden Gate Wax samples (G114) under similar conditions. These metabolites were further expressed in lower concentrations at 21 dpi race 1 samples (G121) while the resistant genotype kept a moderately high production of these metabolites in samples (T121) at 21 dpi. Kaempferol 3-O-rhamnoside-7-O-glucoside, phylloquinone and sennoside D were produced in moderately high concentrations in the resistant genotype Teebus-RR-1 at both 14 and 21 dpi in race 3 infected samples (T3) while these metabolites were suppressed in the susceptible genotype Golden Gate Wax race 3 infected samples (G3) at both time points (Figure 3). The majority of the differentially expressed metabolites in both these cultivars belong to an array of compound classes including flavonoids, terpenoids, fatty acids and phenols that have been found in numerous plant species including legumes (Table 2; Supplementary Table S1).

**TABLE 2:** Putatively identified metabolites that were significantly expressed in common bean genotypes infected with *U. appendiculatus* strains.

No.	rt (min)	m/z	Metabolite name	Molecular formula	Class	*p*-value	References
1	15.77	135.89	Adenine	C_5_H_5_N_5_	Purine	9.9 × 10^−17^	(Ye et al., 2013)
2	11.43	279.28	Graveoline	C_17_H_13_NO_3_	Alkaloid	3.7 × 10^−14^	Ahmed et al. (2022)
3	6.88	284.22	Rhein	C_15_H_8_O_6_	Anthraquinone	1.44 × 10^−15^	Balida et al. (2022)
4	8.03	655.13	Malvin	C_29_H_35_ClO_17_	Anthocyanin	7.8 × 10^−08^	Liu et al. (2022)
5	5.74	191.02	2-Amino-3,7-dideoxy-D-threo-hept-6-ulosonic acid	C_7_H_13_NO_5_	Amino acid	6.4 × 10^−09^	Fu et al. (2022)
6	7.41	302.03	Quercetin	C_15_H_10_O_17_	Flavonoid	3.2 × 10^−08^	Saleh et al. (2019)
7	5.64	546.31	Afzelechin-(4alpha->8)-afzelechin	C_3_H_26_O_10_	Proanthocyanin	0.0004	He et al. (2021)
8	15.72	450.27	Phylloquinone	C_31_H_46_O_2_	Vitamin	0.0003	Vardavas et al. (2006)
9	5.54	316.07	3-O-methylquercetin	C_16_H_12_O_7_	Flavonoid	2.9 × 10^−05^	Kumar et al. (2015)
10	11.79	330.19	Tricin	C_17_H_14_O_7_	Flavonoid	0.0002	Liu et al. (2021)
11	8.46	610.27	Rutin	C_27_H_30_O_16_	Flavonoid	3.2 × 10^−07^	Shun-Cheng, 2012
12	0.77	216.03	Xanthotoxin	C_12_H_8_O_4_	Phenol	7.6 × 10^−08^	Fu et al. (2022)
13	13.38	595.29	Kaempferol 3-O-rhamnoside-7-O-glucoside	C_27_H_30_O_15_	Flavonoid	0.0003	Moloto et al. (2020)
14	13.76	482.26	Daphnetoxin	C_27_H_30_O_8_	Terpenoid	2.4 × 10^−5^	Ohiagu et al., 2021
15	5.5	203.08	(5-Phenyl-1,2,4-triazol-3-yl) urea	C_9_H_9_N_5_O	Energetic salts	0.0007	(Thottempudi and Shreeve, 2011)
16	7.48	580.13	Carlinoside	C_26_H_26_O_15_	Flavonoid	0.0147	Dias et al. (2021)
17	12.67	578.17	Procyanidin B2	C_30_H_26_O_12_	Proanthocyanin	0.0127	(Amarowicz and Troszyńska, 2003)
18	1.24	342.11	D-sucrose	C_12_H_22_O_11_	Flavonoid	9.0 × 10^−6^	Chua et al. (2008)
19	10.48	492.24	Caryoptin	C_26_H_36_O_9_	Terpenoid	0.0035	(Munakata, 1975)
20	5.91	474.12	Agrimophol	C_26_H_34_O_8_	Phenol	0.0041	Le et al. (2018)
21	0.88	632.12	Oleanoic acid 3-O-glucuronide	C_36_H_56_O_9_	Saponin	0.0017	Chung et al. (2020)
22	0.85	378.08	Chlorflavonin	C_18_H_15_ClO_7_	Flavonoid	3.5 × 10^−5^	Wang et al. (2020)
23	0.87	388.11	Tuberonic acid glucoside	C_18_H_28_O_9_	Lipid	0.0004	Ishikawa et al. (2011)
24	10.53	722.32	Kansuinine B	C_38_H_42_O_14_	Terpenoid	0.0049	(Vasas and Hohmann, 2014)
25	5.98	432.1	Vitexin	C_21_H_20_O_10_	Flavonoids	0.0009	Krcatović et al. (2008)
26	12.13	848.51	Sennoside D	C_42_H_40_O_19_	Glycoside	0.0023	Le et al. (2021)
27	15.45	649.12	Uvaricin	C_39_H_68_O_7_	Acetogenins	0.0115	Tempesta et al. (1982)
28	5.22	372.06	Syringin	C_17_H_24_O_9_	Flavonoid	0.0175	Kuo et al. (2015)
29	12.38	560.31	3-(Hexopyranosyloxy)-2-hydroxypropyl (9Z,12Z,15Z)-9,12,15-octadecatrienoate	C_27_H_46_O_9_	Lipid	0.0177	Enciso-Roca et al. (2021)
30	12.45	514.31	Cucurbitacin I	C_30_H_42_O_7_	Terpenoid	0.0172	Balkema-Boomstra et al. (2003)
31	7.98	624.16	Kanokoside D	C_27_H_44_O_16_	Terpenoid	0.0087	Chen et al. (2021)
32	6.77	440.12	24-Methylidenecycloartanol	C_31_H_52_O	Terpenoid	0.0051	Anding et al. (1971)
33	11.41	280.28	Linoleate	C_18_H_31_O_2_	Fatty acid	0.0028	Hughes et al. (2001)

**FIGURE 4:**
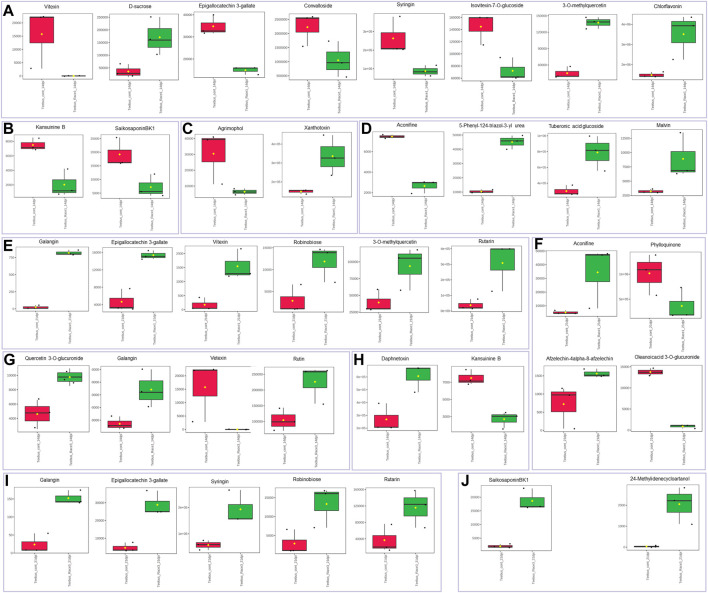
Box plots illustrating concentrations of metabolites differentially enriched in resistant genotype Teebus-RR-1 in response to *U. appendiculatus* race 1 at 14 dpi **(A–D)**, 21 dpi **(E–F)**, race 3 at 14 dpi **(G and H)** and 21 dpi **(I and J)**. **(A)** Flavonoids. **(B)** Terpenoids. **(C)** Phenols. **(D)** Other metabolites belonging to an array of compound classes. **(E)** Flavonoids. **(F)** Other metabolites belonging to an array of compound classes. **(G)** Flavonoids. **(H)** Terpenoids. **(I)** Flavonoids. **(J)** Terpenoids. The red indicates the peak area quantification of metabolites extracted from the control and green indicates the peak area quantification of metabolites extracted from plants infected with *U. appendiculatus*.

**FIGURE 5:**
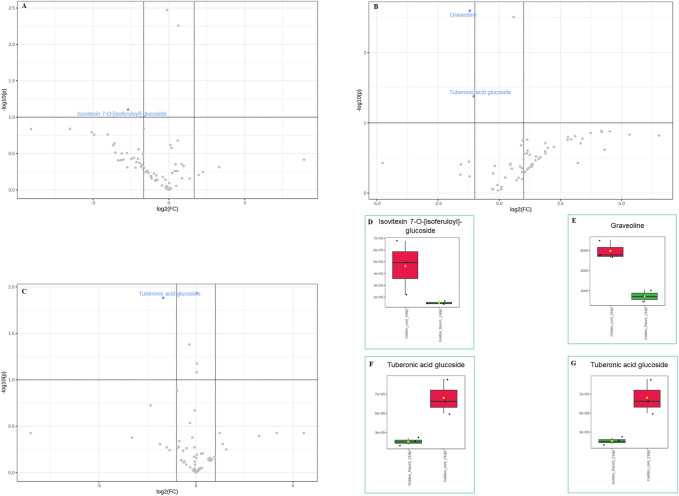
Volcano and box plots showing metabolite changes and concentrations in common bean susceptible genotype Golden Gate Wax response to *U. appendiculatus* race 1 at 14 dpi (A&D—flavonoid), 21 dpi [B, **(E)** alkaloid and **(F)** lipid], race 3 at 21 dpi (C&G—lipid). The blue indicates significantly down regulated metabolites while grey indicates non-significant metabolites in *U. appendiculatus* infected plants. The red indicates the peak area quantification of metabolites extracted from the control and green indicates the peak area quantification of metabolites extracted from plants infected with *U. appendiculatus*.

The resistant genotype Teebus-RR-1 enriched more metabolites in rust infected plants compared to non-infected control samples (Figure 3) for the different treatments. An upregulation of metabolites was prevalent in the resistant genotype Teebus-RR-1 compared to the susceptible genotype that had minimum regulation of metabolites leading to fewer being significant (Figures 5A–C). Metabolites belonging to compound classes such as flavonoids including D-sucrose, 3-O-methylquercetin, chlorflavonin and phenol xanthotoxin (Figure 4A) were enriched in *U. appendiculatus* race 1 infected samples at 14 dpi; while vitexin, isovitexi-7-O glucoside, saikosaponinBK1 and agrimophol were down regulated (Figures 4A–C). Other metabolites belonging to an array of compound classes such as lipids, anthocyanins and energetic salts were also differentially enriched in the resistant genotype Teebus-RR-1 at 14 dpi when infected with *U. appendiculatus* race 1 (Figures 4D,F; Table 2). At 21 dpi, most of the differentially expressed metabolites including flavonoids and other compound classes were up regulated and expressed at exponentially higher concentrations, except for phylloquinone (Figures 4E, F). On the other hand, quercetin, galangin and rutin were up regulated, while vitexin was down regulated in the Teebus-RR-1 infected with race 3 *U. appendiculatus* pathogen at 14 dpi (Figure 4G). Furthermore, terpenoids daphnetoxin, and kansuinine B were differentially expressed at varying concentrations when compared to the control (Figure 4H). In the resistant genotype Teebus-RR-1, flavonoids and terpenoids were expressed at slightly higher concentrations in the *U. appendiculatus* race 3 infected samples at 21 dpi in comparison to control samples (Figures 4I, J).

In the susceptible genotype Golden Gate Wax, isovetexin-7-glucoside was down regulated at 14 dpi with *U. appendiculatus* race 1 (Figures 5A,D), while graveoline and tuberonic acid glucoside were also down regulated at 21 dpi (Figures 5B, C, E–G). There were no differentially expressed metabolites at 14 dpi with *U. appendiculatus* race 3 (Supplementary Figure S3). Meanwhile, tuberonic acid glucoside was differentially down regulated at 21 dpi in *U. appendiculatus* race 3 infected plants (Figure 5C).

### 3.3 Infection with *U. appendiculatus* triggers defence metabolomic pathways

**FIGURE 6:**
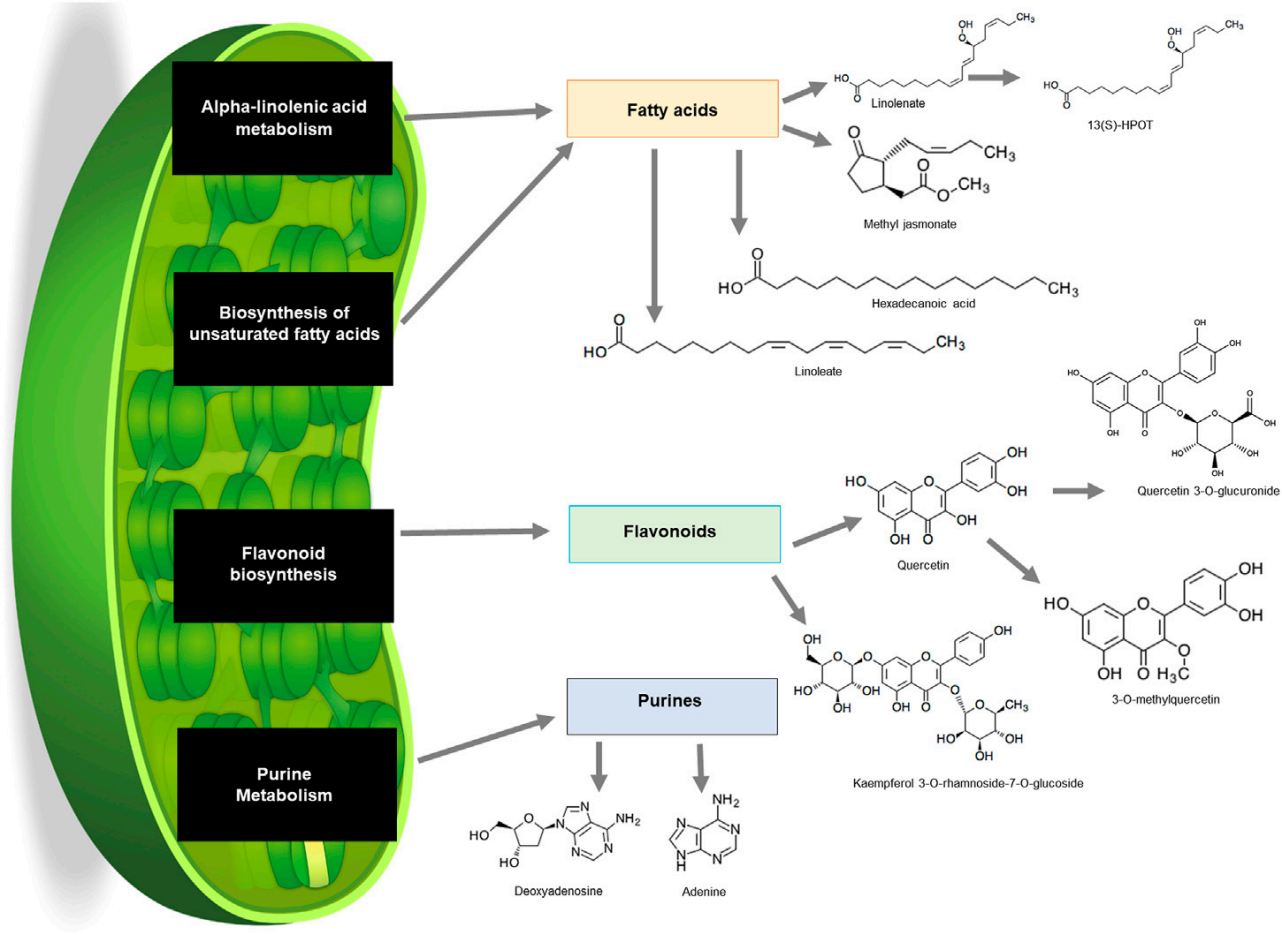
Schematic summary of pathways in common bean plants that contributed to the production of a wide variety of compounds from different classes on KEGG. Fatty acids (orange), flavonoids (green) and purines (blue) from leaf samples.

The KEGG pathway analysis revealed that numerous metabolites that were putatively annotated were associated with several pathways including alpha-linolenic acid metabolism, biosynthesis of fatty acids, flavonoid biosynthesis and purine metabolism (Figure 6). Alpha-linolenic acid metabolism and biosynthesis of unsaturated fatty acids pathway are known to synthesise a group of fatty acids including linolenate, methyl jasmonate, hexadecenoic acid and linoleate. Linolenate is known as a backbone of metabolite 12(S)-HPOT. Meanwhile, flavonoid biosynthesis pathway resulted in the biosynthesis of quercertin from which 3-O-methylquercetin and quercetin 3-O-glucuronide were derived (Figure 6; Supplementary Table S1). Purine metabolism pathway on the other hand was also present and yielded adenine and deoxyadenosine.

## 4 Discussion

### 4.1 Metabolomic analysis of *P. vulgaris genotypes* in response to *U. appendiculatus*

Metabolites are products that play major roles in plant response to environmental stress conditions. Currently, there is limited knowledge on metabolites involved in resistance against an array of fungal and bacterial pathogens in legumes (Makhumbila et al., 2022). In this study, an untargeted metabolome profiling of two common bean genotypes Teebus-RR-1 and Golden Gate Wax were investigated for their response to race 1 and 3 *U. appendiculatus* infections at different time-points (14 and 21 days). The analysis demonstrated that under *U. appendiculatus* infection, different compounds were enriched at varying concentrations in both genotypes at different time points. The resistant genotype showed dominance in metabolite expression in response to *U. appendiculatus* infections when compared to non-infected controls. For instance, 3-O-methylquercetin was highly enriched at 14 dpi when treated with race 1 in the resistant genotype. An increase in 3-O-methylquercetin has been associated with an increase of hydrogen peroxide (H_2_O_2_)) due to biotic stress conditions, which is converted from reactive oxygen species (Quan et al., 2008; Kumar et al., 2015). H_2_O_2_ is a signalling molecule that regulates development and stress adaptation in plants. In addition, Singh et al. (2021) reported that an increase of 3-O-methylquercetin can also be attributed to an increased accumulation of quercetin in resistant genotypes under biotic stress. Quercetin is a flavonoid known to play a role in the process of protecting plants against stress effects. Moreover, quercetin and rutin were also found to be upregulated in the resistant genotype at 21 dpi. Rutin is a flavonoid that plays a key role in protecting plants against pathogens (Kianersi et al., 2020). The increase in flavonoid levels such as quercetin and its derivatives, and rutin among others, have been reported in pathogen-infected plants (Hadrami et al., 2011; Kianersi et al., 2020). In this study, similar results were observed in the Teebus-RR-1 genotype, suggesting the role of flavonoids in defence in response to *U. appendiculatus* at different time-points.

Terpenoids such as daphnetoxin was induced in the resistant genotype at 14 dpi compared to the non-infected control. Daphnetoxin has been reported for its toxicity (Chen et al., 2022), possibly contributing to the suppression of the pathogen growth in the plant. On the other hand, kansuinine B was suppressed in the same genotype at 14 dpi compared to non-infected control. Reduced concentrations of terpenoids have been reported in *Chrysanthemum morifolium* inoculated with *Alternaria tenuissima* (He et al., 2022). The decreased concentration of terpenoids have been shown to cause cell membrane damage as they merge with phosphor-lipid acyl chains (Hammerbacher et al., 2019). In addition, an alkaloid such as aconifine was also moderately enriched in a resistant *U. appendiculatus* treated genotype (Figure 3). Generally, alkaloids have been reported for their role in herbivory defence in plants (Macel, 2011; Bhambhani et al., 2021), suggesting their possible role in common bean response to *U. appendiculatus*.

On the other hand, the susceptible Golden Gate Wax genotype differentially expressed compounds such as alkaloids and lipids at different time-points post infection with rust races 1 and 3. Metabolites that were downregulated in the susceptible genotype in response to *U. appendiculatus* races included tuberonic acid glucoside (Figure 5). The downregulation of lipids can be linked to lipid metabolism degradation in plants infected with pathogens, therefore signalling the genotypes’ susceptibility (Robison et al., 2018; Foucher et al., 2020). In addition, *U. appendiculatus* slows down photosynthetic activity by forming pustules on susceptible genotypes, as it was observed in our study (Figure 2H), consequently causing the cell wall to collapse by lipid peroxidation (Bojtor et al., 2019). An exploration of other metabolites in the susceptible genotype indicated a dramatic decrease in the expression of phenolic compounds (xanthotoxin and chlorogenic acid), which are essential in the plants defence against *U. appendiculatus* (Omara et al., 2022). The pathogen *U. appendiculatus* could be promoting the synthesis of proteins for feeding that are synthesised from an array of pathways including lipids (Link, 2020). This group of metabolites were downregulated in the susceptible genotype in our study.

It has been widely reported that exposure of plants to pathogen stress increases the production of flavonoids, terpenoids and phenols as a defence strategy (López-Gresa et al., 2010). Flavonoids signal compounds in plant-pathogen interaction that also tend to be highly produced in resistant as compared to susceptible genotypes (Steinkellner et al., 2007; Chin et al., 2018). Terpenoids influence the plant’s ability to inhibit pathogen attack (Toffolatti et al., 2021). Phenols protect plant tissues from the toxic effects of pathogens (Kumar et al., 2015), while alkaloids store nitrogen reserves during pathogen attack (Ali et al., 2019).

The results of this study also show that due to *U. appendiculatus* infection, compounds such as D-sucrose, 3-O-methylquercetin, phenol xanthotoxin and chlorflavonin were highly enriched in the resistant genotype compared to the susceptible genotype. A recent study reported similar findings that resistant genotypes tend to secrete a larger number of metabolites at high concentrations when compared to susceptible genotypes under disease pressure (Robison et al., 2018). Additionally, in barley genotypes infected with stripe rust, similar patterns of metabolite upregulation in resistant genotypes were reported (Singla et al., 2020).

### 4.2 Defence strategies of common bean plants to *U. appendiculatus*

In this study, we found that chlorflavonin, D-sucrose, xanthotoxin, tuberonic acid glucoside, malvin, vitexin, robinobiose, 3-O-methylquercetin, rutarin, aconifine, galangin, rutin, daphnetoxxin, syringin and 24-Methylidenecycloartanol were highly enriched metabolites in response to *U. appendiculatus* infection with aconifine being the most enriched (Figure 4F). Aconifine is an alkaloid that has been widely linked to *Aconitum karakolicum Rapaics* which is an antiproliferative that prohibits pathogen cell growth (Tashkhodjaev and Sultankhodjaev, 2009; Thawabteh et al., 2019). On the other hand, rutarin was also expressed in abundance at 21 dpi when the plant was under rust infection. The compound has been reported to have antifeedant/antiparasitic properties in tree plants (Lacroix et al., 2011). Flavonoid D-sucrose was also enriched in common bean at 14 dpi in the resistant genotype. Certain studies have highlighted the importance of this metabolite in plant growth and associated the metabolite with fungal pathogen virulence in maize (Wahl et al., 2010). In another study, it was highlighted that the synthesis of sucrose is beneficial for the plants overall growth and tolerance to stressors (Stein and Granot, 2019). Xanthotoxin, a phenolic compound was also differentially expressed in plants infected with the *U. appendiculatus* pathogen. This compound has been appraised for its ability to interact with free radicals and biological molecules that might cause lipid peroxidation, protein damage, enzyme inhibition and genetic oxidation that might result in cell death (Bajerová et al., 2014; Dumanović et al., 2021). Another antioxidant characterized compound that was differentially expressed was galangin that has been found to be a beneficial antioxidant in red kidney bean evaluated under water deficiency (Manoj et al., 2022). This is in concurrence with our finding that certain metabolites are highly enriched in common bean leaves as a stress defence strategy. However, it is still unclear how metabolites interact with genes expressed, and for this reason, an integrated omics study to unravel underlying response mechanisms involved in common bean rust interactions will have far reaching impact in common bean breeding.

### 4.3 Metabolomic pathways contribute to the plants ability to respond to *U. appendiculatus* attack

Fatty acids that are synthesized by the plant tend to undergo prokaryotic or eukaryotic pathway. During this occurrence, they are converted to complex lipids for assembly in the eukaryotic pathways. This consequently leads to the production of plant fats that contribute to the plants overall nutrition (Chellamuthu et al., 2022). In the current study, metabolites synthesized through the alpha-linolenic acid metabolism and biosynthesis of unsaturated fatty acids were not significantly enriched. This possibly indicates that despite stress conditions, other metabolomic activities responsible for the overall productivity still occur. Interestingly, Kumaraswamy et al. (2011) reported a 4-fold expression of linolenic acid in barley resistant genotypes infected with *Fusarium graminearum* compared to the susceptible genotypes.

In terms of flavonoids, they have been widely reported for their contribution in plant biotic and abiotic stress response (Jan et al., 2022). Our results found that quercetin, rutin and galangin were up regulated in the resistant genotype infected with *U. appendiculatus* at both time points. Similar findings reported quercetin and kaempferol derivatives to be effective contributors to plants stress response because of their antioxidant properties (Koskimäki et al., 2009). The expression of flavonoids in the susceptible genotype (Golden Gate Wax) was lower when compared to the resistant, suggesting reduced flavonoid biosynthesis. This is supported by the study of Nagamatsu et al. (2007) who reported that reduced flavonoid biosynthesis in soybean under viral pathogen stress may be associated with cell wall degradation. Similarly, purine metabolism has also been appraised for its contribution to stress adaptation by contributing to nitrogen metabolism (Watanabe et al., 2014). However, metabolites belonging to the purine metabolism pathway were not significantly enriched in the current study. The biosynthesis of metabolites in plants is related to genes (Raza, 2020) and thus complex, therefore metabolomics and transcriptomics studies should be integrated to gain insight on the integral pathways that play a role in defence.

## 5 Conclusion

In the present study, the whole metabolome of common bean infected with *U. appendiculatus* was characterised. The differences in terms of metabolites between the inoculated and non-inoculated common bean showed that flavonoids, terpenoids, alkaloids and others play an important role in defence response to *U. appendiculatus* infection. Flavonoid pathway was the main defence response in common bean. Further investigations on early response (24–72 h post-infection) will give a clearer picture of the metabolites involved at the initial stages of infection. Metabolomic expression patterns of genotypes at early and late infection stages can be used to understand plant-pathogen interactions and significantly upregulated metabolites can serve as biomarkers in breeding programmes. Overall, this study provides new insights in understanding common bean interactions with *U. appendiculatus*. Future research studies should focus on integrating metabolomics with other OMICs to uncover possible underlying defence mechanisms which could represent a helpful tool for developing common bean resistant varieties toward *U. appendiculatus*.

## Data Availability

Metabolomic data presented in the study are deposited in the MetaboLights repository, accession number MTBLS6972.

## Supplementary material

SUPPLEMENTARY TABLE S1 A list of all putatively identified differentially expressed metabolites expressed across common bean groups evaluated under *U. appendiculatus* stress.

SUPPLEMENTARY FIGURE S1 The PCA score plot of *P. vulgaris genotypes with varying resistance to U. appendiculatus* races.

SUPPLEMENTARY FIGURE S2 The OPLS- DA scores plot of *P. vulgaris* genotypes with varying resistance to *U. appendiculatus* races.

SUPPLEMENTARY FIGURE S3 Volcano plot showing none significant metabolites in susceptible genotype Golden Gate Wax infected with *U. appendiculatus* race 3 at 14 dpi.
