# Supplementary figures and images for: Metabolome profile variations in common bean (Phaseolus vulgaris L.) resistant and susceptible genotypes incited by rust (Uromyces appendiculatus)
Source: Front Genet. 2023 Mar 16;14:1141201. doi: 10.3389/fgene.2023.1141201 (PMC10060544; doi:10.3389/fgene.2023.1141201)

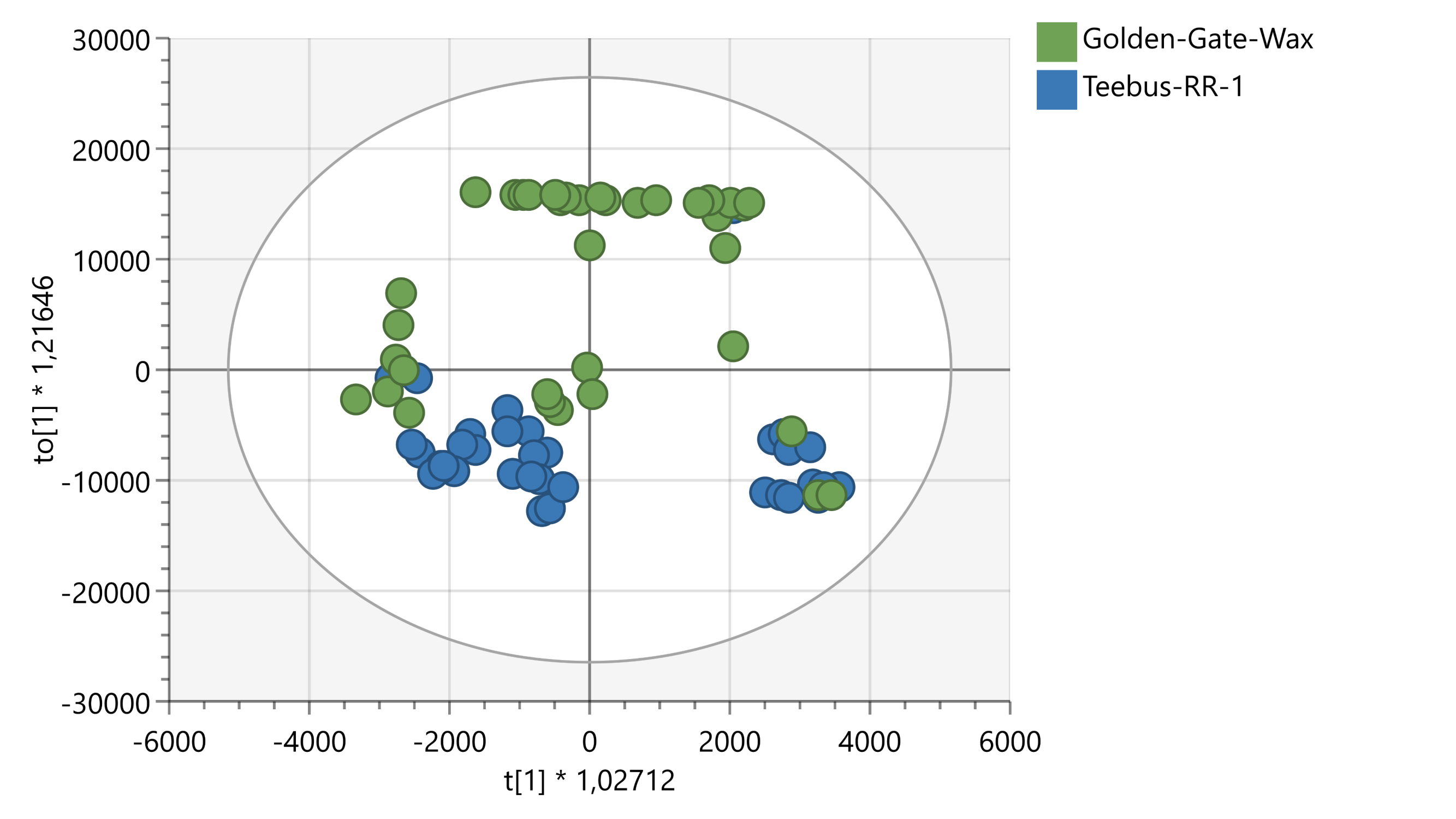

Supplement: Supplementary file 1 [file Image3.TIF]

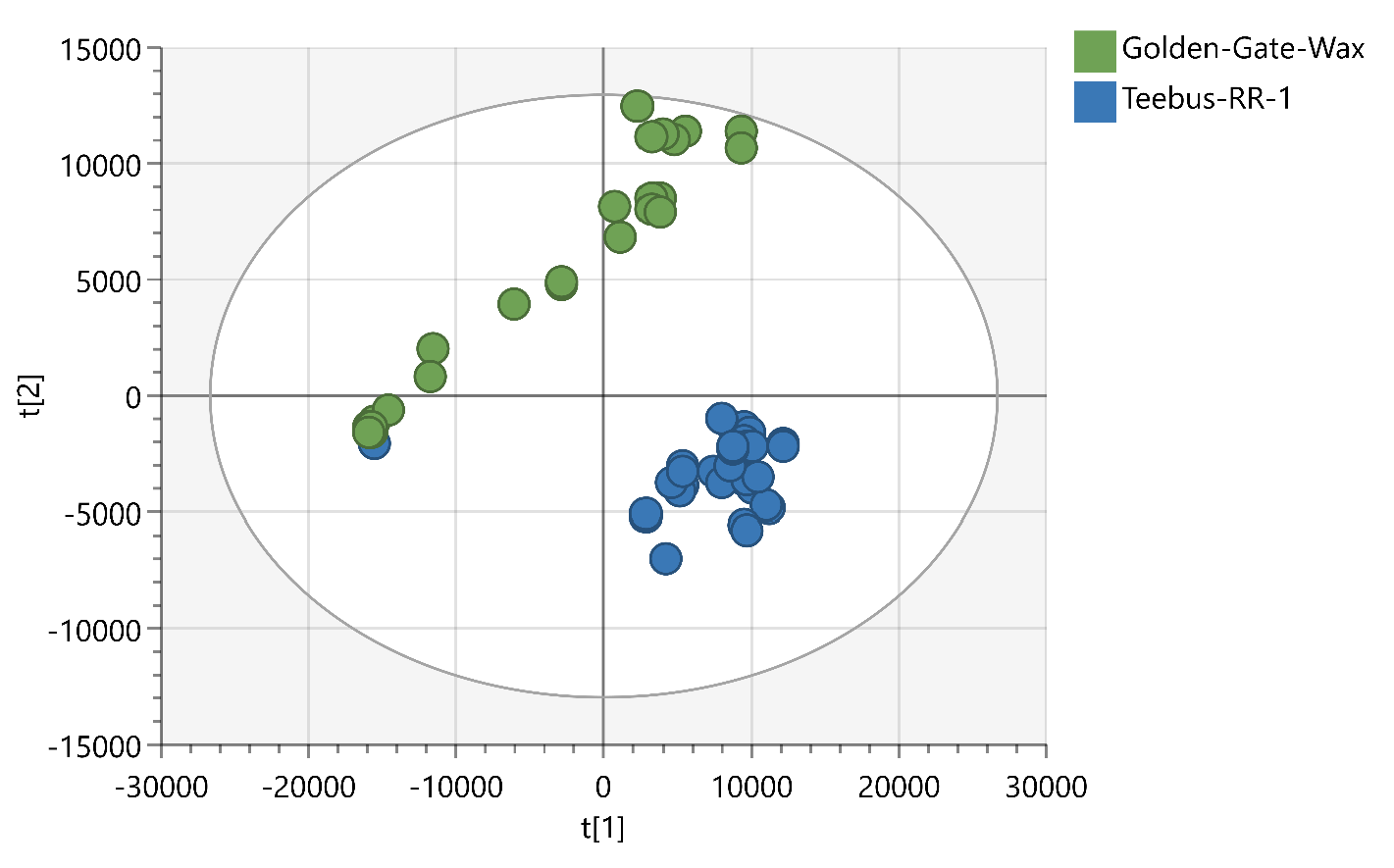

Supplement: Supplementary file 2 [file Image2.TIF]

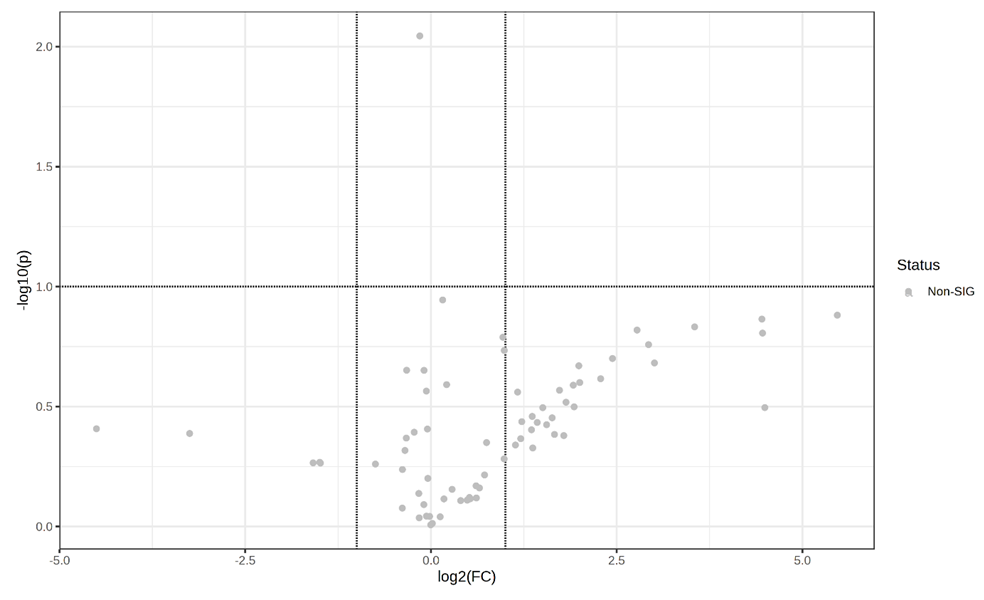

Supplement: Supplementary file 3 [file Image1.TIF]
